# Influence of Hydration and Temperature on the Na_x_CO_2_ Based Transducer Voltage

**DOI:** 10.3390/mi15111334

**Published:** 2024-10-31

**Authors:** George-Claudiu Zărnescu, Esmaeil Jalali Lavasani, Lucian Pîslaru-Dănescu, Ioan Stamatin

**Affiliations:** 1 Laboratory of Sensors/Actuators and Energy Harvesting, National Institute for Research and Development in Electrical Engineering ICPE-CA, 030138 Bucharest, Romania; george.zarnescu@icpe-ca.ro; 2 3Nano-SAE Research Center, Faculty of Physics, University of Bucharest, P.O. Box MG 38, 077125 Bucharest-Magurele, Romania; esmaeil.jalali@3nanosae.org (E.J.L.); istarom@3nanosae.org (I.S.)

**Keywords:** sodium cobalt oxide, hydration, thermomagnetic effect, thermoelectric effect, energy harvesting, transducer

## Abstract

This paper presents an experimental approach to maximizing the voltage generated by Na_x_CoO_2_ and improving the overall efficiency of the p-type thermoelectric leg by doping with Na up to x = 0.88. Two samples with different geometries were tested, each measured with and without an additional magnetic field applied in the direction of the temperature gradient. The properties of sodium cobaltite in response to hydration were explored, at temperatures between 300 and 380 K. Water injection boosted the current and power up to 75–100 µW at a temperature of 350–360 K. This power boost can be attributed to an electron-ion fluid flow pattern maintained by the longitudinal thermomagnetic effect and by water molecules forming hydrogen bonds with oxygen atoms in the CoO_2_ layers, inside the material. An electronic circuit was designed to boost the voltage to the desired level, for three or more sodium cobaltite samples mounted in parallel, and to store the energy in a supercapacitor. The output voltage and resistivity change of sodium cobaltite samples can be readily used as a humidity and temperature-sensing element in a transducer when paired with an appropriate electronic conditioning scheme.

## 1. Introduction

Sodium cobaltites (Na_x_CoO_2_, with 0.3 < x < 1) are versatile p-type semiconductors with a wide range of applications. These materials are used in applications like supercapacitors, superconductivity, water oxidation electrocatalysis, and sodium-ion batteries. Additionally, they can serve as transducers for thermal energy harvesting and as sensing elements for environmental monitoring, which is the focus of this paper. The thermoelectric performance of Na_x_CoO_2_ varies with sodium concentration. Poor thermoelectric properties are observed in the 0.1 < x < 0.35 doping region, while the 0.5 < x < 0.60 region exhibits stability and primarily ferromagnetic characteristics [1]. Antiferromagnetic properties dominate in the range 0.62 < x < 0.67, displaying normal-range thermoelectric effects (Seebeck coefficient of 100 µV/K). In the 0.75 < x < 0.9 range, there is emergence of long-range magnetic ordering with polaron formation, and the material exhibits metallic behavior with decreasing Curie–Weiss temperatures and ferromagnetic couplings [2,3].

J. Sugiyama [4] observed that the thermoelectric properties of Na_x_CoO_2_ significantly improve for x = 0.75…0.9 samples when compared to x = 0.6…0.65 samples, down to 2 K. Although at 300 K the resistivity of samples with x = 0.9 was higher by one order in magnitude than that of Na_0.6_CoO_2_, a strong magnetic field can induce a large negative magnetoresistive effect [5] by changing the electrons’ unordered state back to a magnetically ordered state, similar to the metallic behavior of samples measured at low temperatures.

Beyond a Na content of x = 0.9…1, the material assumes insulating properties, with a sharp decline in the power factor attributed to increased resistivity [6,7].

Recent studies, including those of Namhongsa et al. [8] and Yin [9], provide evidence that under a magnetic field, the thermopower coefficient of sodium and calcium cobaltites is suppressed at low temperatures, between 20 and 120 K. Suppression of thermopower is linked to a low entropy state. As charge carrier concentration and mobility increase with temperature and the applied magnetic field, the electronic band structure is modified and spin-splitting effects are induced in the sample structure. All the above-mentioned effects contribute to an enhanced thermoelectric power factor. Also, for the poorly explored Na 0.75 < x < 0.86 high-doping region, the power factor was further enhanced. At a critical sodium doping of x = 0.85, the power factor and figure of merit (ZT) were increased by 40 times (at 80 K) [10]. Because the layered crystal of Na_x_CoO_2_ has stacks of CoO_2_ 2D triangle sheets and Na layers, as sodium doping increases in that interval, the thickness of the CoO_2_ layers gradually increases. This increased layer thickness should lead also to better water autoionization, producing hydronium ions H_3_O^+^ and hydroxide ions. Hydroxide ions typically attach to Na^+^ ions, while hydronium ions are intercalated between CoO_2_ layers, replacing the expelled Na^+^ ions.

Additionally, Richard Dronskowski, Ichiro Terasaki et al.’s book chapter [11] and Xiaofeng Tang’s comprehensive study [12] on Na_x_Co_2_O_4_ as a promising thermoelectric oxide material for high-temperature power generation applications offer valuable insights, highlighting superior synthesis techniques, crystal structures, and thermoelectric properties, including high ZT (performance coefficient) values [12]. Both the book chapter and research paper provide a crucial context for our investigation, particularly in understanding the roles of spin entropy and superconducting behaviors in augmenting the thermoelectric properties of similar compounds. In contrast to spin entropy theory, which makes correlations for individual sites, the spin wave scenario accounts for electron correlations between adjacent sites. The impact of spin waves on thermopower can be explained by the relationship involving entropy, from the disordering of spin orientations throughout the entire lattice. The number of spin wave excitation modes is influenced by both temperature and magnetic field. This dependence can shape thermoelectric power, and electrical and thermal conductivity [9,11,12].

The exfoliation process of metal oxides has been investigated to produce nanoflakes or nanosheets. These ultrathin layers can be used in supercapacitors or batteries. Although in ACoO_2_ metal oxides, where A is the ion to be used in the exfoliation process, LiCoO_2_ was widely used, it has been observed that even by replacing Li by Na, Rb, Cs, the gap between the valence band and the conduction band remains intact. There is electrostatic bonding between negative transition metal oxide layers and the positive Li-ion or Na-ion layer. These bonding examples are for LiCoO_2_ and NaCoO_2_. Exfoliation of these materials was not straightforward but has recently become possible by using chemical ion replacement reactions, such as replacing Li or Na by H. A question also arises about the 2d CoO_2_ layers, as to whether they can be spaced further apart by increasing the size of intercalation ions, like hydronium ions H_3_O^+^ intercalation. This case has been highly debated because superconductivity has been claimed to occur inside CoO_2_ layers, because the triangular lattice of Co spins was highly frustrated by this intercalation of ions. However, understanding is still lacking as to how CoO_2_ layers can be manipulated to achieve superconductivity at higher temperatures [13]. The Na_x_CoO_2_ energetics were analyzed by exploring the temperature dependence of their catalytic activity. Linear sweep voltammetry curves were recorded from 298 to 318 K [14]; these graphs show that oxygen evolution reaction activity improves with elevated temperature. Also, these curves indicate that activation energy is thermodynamically sensitive with temperature. From all Na_x_CoO_2_ samples (x = 1.0, 0.9, 0.75, 0.5), Na_0.75_CoO_2_ displays the best oxygen reduction performance, being the best candidate for electrocatalysis [15]. The substitution of Co by 10 mol% solution of Cr, Ni, Zn, W or Bi leads to a decrease in the values of electrical resistivity of Na_0.55_Co_0.90_M_0.10_O_2_ ceramics, where M is the transition or heavy metal, and an increase in sample thermal diffusivity, thermal conductivity, and the Seebeck coefficient values. The maximum Seebeck coefficient values were around 300 μV/K at 600 K and between 600 and 670 μV/K at 1073 K for Bi and W doped samples [16]. It was observed that calcium Ca_3_Co_4_O_9_ and sodium Na_0.75_CoO_2_ cobaltites have complementary advantages. While calcium cobaltites are stable at high temperature and humidity levels, sodium cobaltites degrade more rapidly. The intergrowth of sodium and calcium cobaltite layers should increase the long-term stability of these thermoelectric materials. Moreover, a low percent 0.05% of dopants like Na or Ba instead of Ca ions for the calcium cobaltite Ca_3_Co_4_O_9_ can increase the Seebeck coefficient, electrical conductivity, the power factor, and figure of merit [17,18]. If the percent of dopants exceeds 0.1%, the carrier concentration increases but the Seebeck coefficient decreases by half.

Considering that for sodium cobaltite a doping interval of 0.85–0.88 was leading to the highest observed power factor at 80 K [10], the first experimental approach in this paper was to investigate the higher doping x = 0.88 and to experiment with a higher temperature interval, between 270 and 370 K. This temperature interval has been poorly explored by researchers, mainly because sample resistivity increased with temperature. Minhyea Lee, Liliana Viciu et al. [10] concluded that all analyzed samples had a peak value for the Seebeck coefficient at 130 K. But it can be noted for the sample with number 5, with x = 0.88, that the Seebeck coefficient was still high at 300 K, over 210 μV/K. Another experimental approach was to explore methods to decrease sample resistivity between 300 and 370 K without sacrificing the Seebeck coefficient. An efficient way was to use two strong NdFeB magnets with N35–N37 magnetization, or with a 1.22 T remanent magnetic field, above and below the sample to induce a large negative magnetoresistance effect, which was amplified by the increasing axial temperature gradient. Furthermore, by adding water to the samples, combined with exposure to negative magnetoresistance and to the axial thermomagnetic effect, conductivity and carrier transport were significantly enhanced, thus improving overall power generation of devices operable within a temperature range of 270 to 370 K.

In the second chapter, the synthesis of Na_x_CoO_2_ material and the design of the transducer are briefly presented. The axial or longitudinal (applied along the temperature difference) magnetic field inside the sample was simulated using FEMM 4.2 software. In the third chapter, comparison graphs illustrate the variation of current and power with temperature and the applied magnetic field. The electrical power variation with temperature graph showed a significant increase in power when an axial magnetic field was applied to the sample. The experimental setup for measuring dry or hydrated Na-cobaltite samples is also described. Additionally, the third chapter explores the combined influence of water and magnetic fields to further boost the current and generated electrical power output. The combined influence on the probe results from three interactions. An ionic current is generated from Na^+^ ions’ movement, the de-intercalation of Na^+^ and the intercalation of hydronium ions, when water is added. Electrical conductivity is increased by the magnetic field and then by water injection. Sample voltage is increased due to both thermomagnetic and thermoelectric effects. An electronic circuit comprising a Meissner oscillator, a voltage regulator, and an astable timer circuit was developed to amplify and control the voltage. This circuit can either charge a supercapacitor or be used as a transducer conditioning module, without the supercapacitor regulating voltage part. Finally, the output signal will provide information about humidity, temperature, or magnetic field strength.

## 2. Materials and Methods

### 2.1. Materials Synthesis and Transducer Design

The Na_x_CoO_2_ materials were prepared using sol-gel and self-combustion synthesis. The polymer-like Na_x_CoO_2_ materials were shaped into pellets and sintered at approximately 970 K in quartz closed jars with a quantity of sodium salts (carbonates) to compensate for sodium desorption from the initial structure and achieve the desired composition of Na_x_CoO_2_. Initially, two different samples were compacted under 3–4 tons of pressure to form pellets. One was 10 mm in diameter and 10 mm thick, while the other was 30 mm in diameter and 2.5 mm thick.

Due to geometric and mechanical strength constraints, as well as superior material properties, sodium cobaltite samples with a diameter of 30 mm and a thickness of 2.5–3 mm were ideal for this study. Geometry is limited by the availability of NdFeB magnets and the mechanical stress resistance of the samples. In the market, larger disk magnets are available with diameters ranging from 27 cm to 42 cm and magnetizations from N35 to N42 at those sizes. The thickness of the sample was 2.5–3 mm because such a device should not operate at temperatures higher than 380 K, when the water is added.

The magnetic field strength at the center of the sample (from the magnets) will not fall below 0.1 T if the magnets are kept close to each other, approximately 4–5 mm apart.

**Figure 1:**
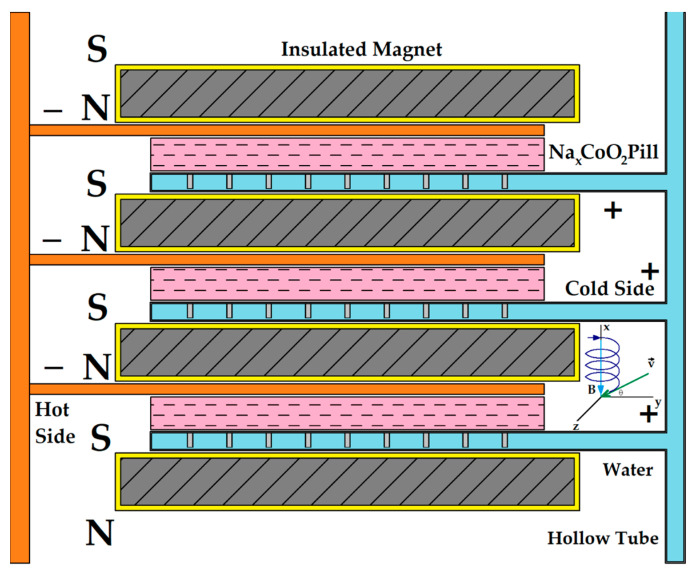
Na_x_CoO_2_ transducer made of three or more samples. The structure resembles a battery with parallel electrical connections that also serve to cool and heat sodium cobaltite pills.

Also, this thickness and surface area amplify the ionic current and Na+ ions’ mobility that is present in the sample. When sodium content decreases, electrical conductivity increases, according to [19]. Figure 1 describes the magnets’ arrangements and sodium cobaltite samples’ parallel connection with the cold and hot side. The hot and cold sides can be made of aluminium or copper to assure both good thermal conductivity and electrical connections; in this case, aluminium was used. Moreover, the cold side round surface could be provided with small fins or inner channels for better cooling. Channels and hollow tubes could be used for the cold side to circulate water and hydrate the pills to form a better thermal energy-harvesting device.

Although the Na^+^ ions’ mobility is low, $13\;{cm}^{2}/(V\cdot s)$, electron mobility can exceed $1000\;{cm}^{2}/(V\cdot s)$ in sodium cobaltite samples with high resistivity [16], similar to semiconductors, depending on the number of dopants. Notably, electrons move in a spiral from the hotter to the colder side due to the presence of a parallel magnetic field. Velocity and mobility are associated with the electric field created by the temperature difference from the drift current. The parallel magnetic field (relative to the generated electric field) and temperature difference are always linked to the longitudinal thermomagnetic coefficient. This effect is similar to, but should not be confused with, the Nernst effect, where the magnetic field is transverse to the temperature gradient.

**Figure 2:**
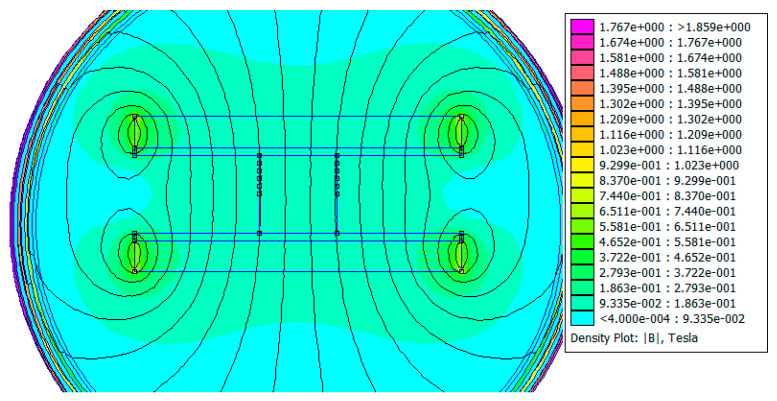
Magnetic field (FEMM) simulation of a sodium cobaltite sample, 10 mm in diameter and 10 mm thick, placed between two (1 mm in thickness) aluminium plates and two NdFeB magnets, 42 mm in diameter and with N35–N37 magnetization.

A 2D FEMM simulation was realized to find out the magnetic field distribution inside the Na_x_CoO_2_ 10 mm-in-diameter sample. The sample thickness was 10 mm and the thickness of each aluminium plate was 1 mm. See Figure 2.

The probe diameter was set to 10 mm. As can be seen from Figure 2, the two NdFeB magnets with a thickness of 4 mm were separated by a distance of 12 mm. From the magnetic field distribution, a magnetic field of 0.0933 T was observed inside the sample and a higher than 0.12 T magnetic field was noted at 6 mm distance from the magnets’ edges. A seven-layers Dirichlet boundary was applied for both simulations.

**Figure 3:**
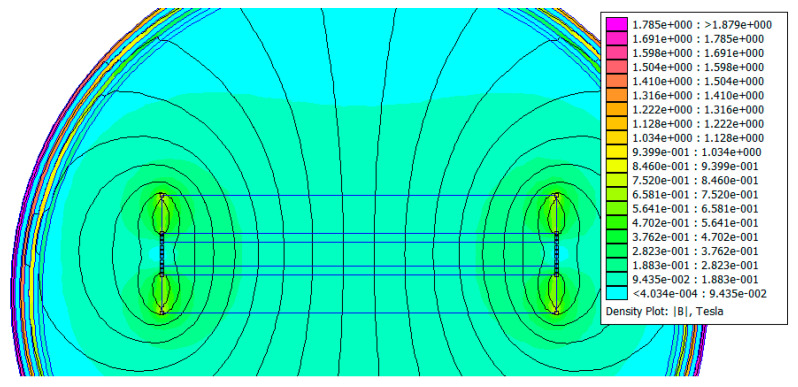
Magnetic field (FEMM) simulation of a sodium cobaltite sample, 30 mm in diameter and 2.5 mm thick, placed between two (1 mm in thickness) aluminium plates and two NdFeB magnets, 42 mm in diameter and with N35–N37 magnetization.

A second 2D FEMM simulation was realized to ascertain the magnetic field distribution inside the Na_x_CoO_2_ 30 mm-in-diameter sample. Sample thickness was 2.5 mm and the thickness of each aluminium plate was 1 mm. See Figure 3.

Probe diameter was not essential for the simulations. As it can be seen from Figure 3, the two NdFeB magnets with a thickness of 4 mm were separated by a distance of 4.5 mm. From the magnetic field distribution, a magnetic field of 0.188 T was observed inside the sample and a higher than 0.282 T magnetic field was noted at the magnets’ and pills’ edges. The seven-layer Dirichlet boundary ensured good accuracy for the magnetic field distribution calculus. Even if we replace the NdFeB N37 grade magnets with the strongest ones, N55 grade (1.5 T remanent magnetic field), the average magnetic field will be around 0.25 T inside the sample with 2.5 mm thickness. Magnets with a remanent magnetic field of 1.5 T are rare and not widely available. In conclusion, the simulations in Figure 2 and Figure 3 show that an average magnetic field of 0.1 T passes through the 10 mm thick sample, while an average field of 0.2 T passes through the 2.5 mm thick sample.

### 2.2. Experimental Setup for Measurements

**Figure 4:**
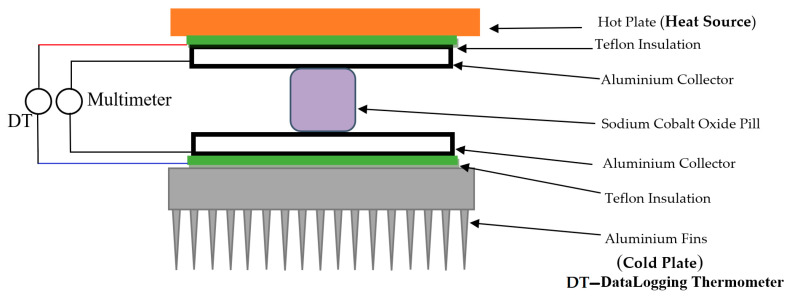
The thermoelectric sample mounted in the electrical heat-press, between two 1 mm thick aluminium collectors.

Figure 4 illustrates the experimental setup, consisting primarily of a heat press, a datalogging thermometer, model SDL200 EXTECH (DT) with four channels for precise temperature measurement of hot and cold plates and sample surfaces, and an R&S HMC8012 digital multimeter with electrical measuring cables connected to the aluminium collectors.

On each side of the sample, aluminium plates were used as collectors, separated from the heat press plates by a thin Teflon layer acting as an insulator. The temperature was set to 380 K to explore the relationship between temperature difference and the sample’s thermoelectric properties. Upon reaching the desired temperature, the heater was turned off, and voltage, current, and electrical resistance were recorded every 10 s as the temperature decreased.

The same mounting procedure was followed to determine the magnetic field’s influence on the dry or hydrated Na_x_CoO_2_ sample. In this case, two magnets were attached, as shown in Figure 1 and Figure 3, positioned 4.5 mm apart, below and above the aluminium collectors. When the temperature reached 380 K, the heater was turned off, and voltage, current, and electrical resistance were recorded every 10 s as the temperature decreased. The natural convection heat sink was placed under the second magnet, disposed under the cold side, to keep a strong magnetic field inside the sample. The probe was manually hydrated with a syringe and then placed in the electrical heat-press; the optimum dosage was between 1.5 and 2 mL to obtain a maximum voltage (0.24 V at 380 K) and current response of over 1 mA. When the sample is hydrated, some of the Na^+^ (at a rate of 0.01/mL) ions are replaced in the structure with H_3_O^+^ ions, while the Na^+^ ions are linked to OH^−^ autoionized water molecules. All H_3_O^+^ ions that are replacing Na^+^ ions are connected within CoO_2_ interlayers [18,20,21].

## 3. Results

### 3.1. Thermoelectric Measurements

Na_x_CoO_2_ exhibits an anisotropic magnetoresistive effect or simply magnetoresistance (MR); when the magnetic field is applied axially (parallel), the MR remains negative. For the radial or perpendicular magnetic field, the ferromagnetism is present in the first case and is suppressed so that the MR changes from negative to positive. When x≥0.9, the material is no more ferromagnetic and has no Curie temperature or hysteresis effect with the changing temperature, becoming paramagnetic.

The strong electron to electron influence and the triangular lattice frustration, which induct a narrow band when a magnetic field is applied, are linked to the large thermoelectric power and to the superconductive state behavior when water is added. This ceramic material can be considered a Mott insulator or a strongly correlated material when x≥0.9. A phase transition in the material occurs around 350 K. Blangero et al. suggest that Na^+^ interlayer redistribution between CoO_2_ layers should be the cause of this phase transition [22]. The Na^+^ interlayer redistribution could also lead to a large thermoelectric power when a magnetic field is applied.

Other dopants can lead to a large thermoelectric power. Polycrystalline samples with a base sodium content of 0.5–0.87, doped with elements such as calcium (Ca), bismuth (Bi), strontium (Sr), and (Cu) up to levels of 0.1 or 10%, exhibit a higher Seebeck coefficient and power factor than undoped sodium cobaltite [16,23,24,25,26]. When dopants such as Sr or Ca are incorporated into the Na layer, they decrease disorder and increase the overall crystallinity of the system. As a result, the mean free path of the free carriers increases, which enhances both their mobility and the probe’s conductivity [27], ultimately raising the figure of merit (ZT) [23]. The same effect is expected when the sample is hydrated and exposed to a magnetic field.

**Figure 5:**
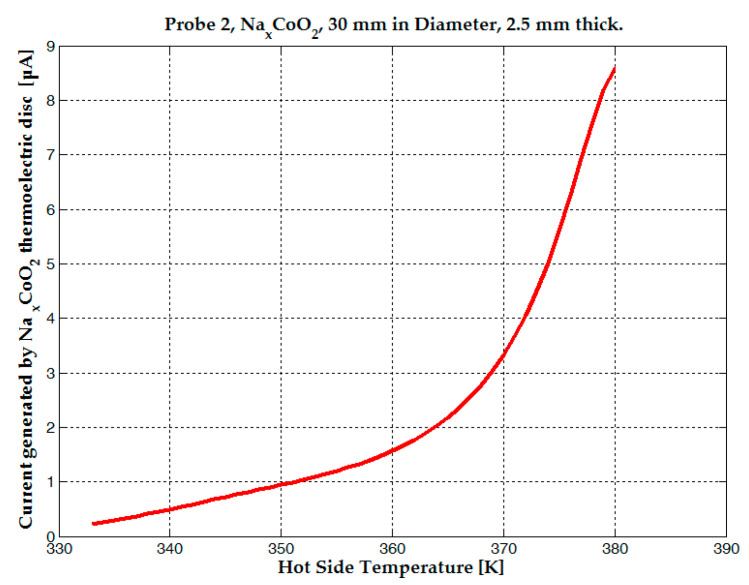
The current generated by the Na_x_CoO_2_ thermoelectric sample under the influence of magnetic field.

The 30 mm-diameter probe exhibited a resistance of 1.9 MΩ at 330 K without a magnetic field, dropping to 380 KΩ when magnets were positioned above and below the pill. Correspondingly, the sample current increased from 0.14 µA to 0.68 µA. At 340 K, resistance was 1.65 MΩ without the magnetic field, falling to 240 KΩ with the magnets, and the current rose from 0.28 µA to 1.9 µA. See Figure 5. Measured voltage was 0.26 V at 330 K and 0.45 V at 340 K, showing a 0.19 V increase over a 10 K temperature rise [28]. The 30 mm-diameter probe magnetoresistance was five to seven times lower than the resistance measured without magnets.

**Figure 6:**
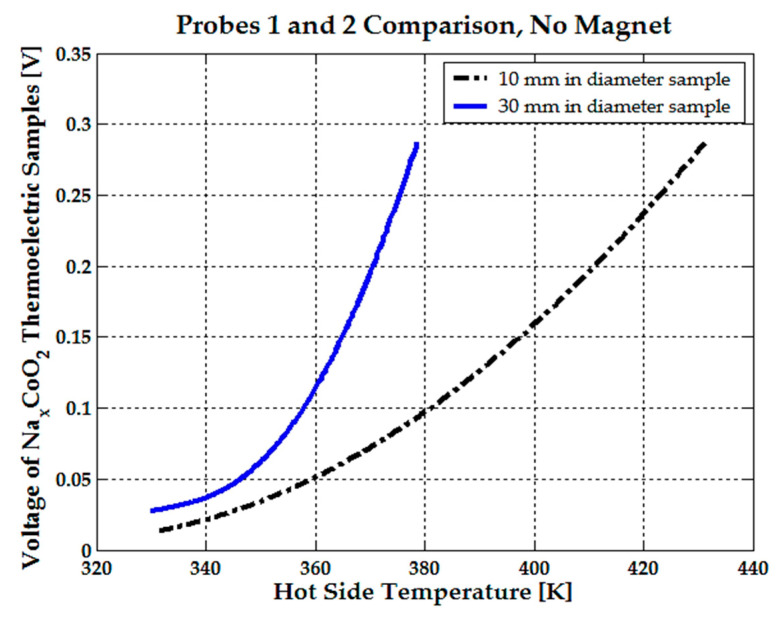
Voltage comparison between the 30 mm-diameter and 10 mm-diameter Na_x_CoO_2_ thermoelectric samples, with no magnets attached.

**Figure 7:**
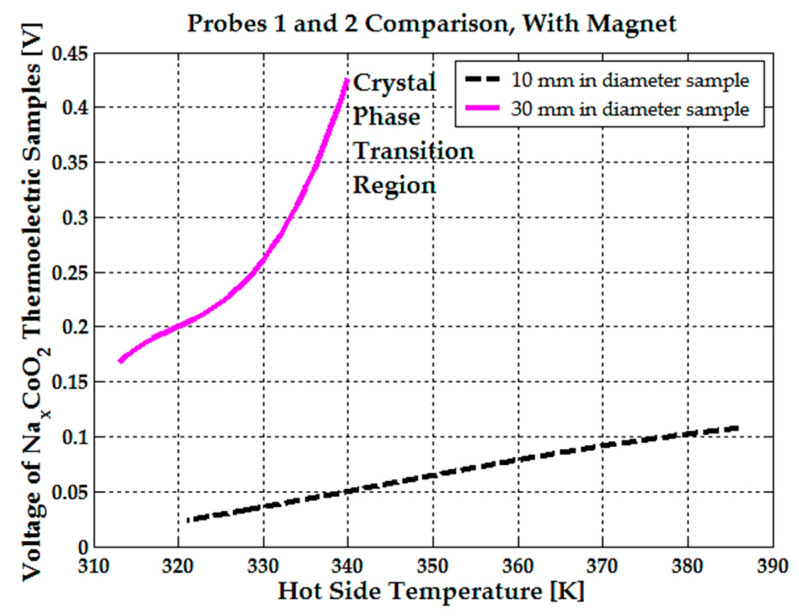
Voltage comparison between the 30 mm-diameter and 10 mm-diameter Na_x_CoO_2_ thermoelectric samples, with NdFeB magnets attached.

A maximum current of 1 µA was observed for the 10 mm-diameter probe, while a current of up to 3.5 µA was measured for the 30 mm-diameter sample, both at a hot-side temperature of 375 K without a magnet attached. When the same NdFeB magnets were used, an average magnetic field of 0.1 T was applied to the 10 mm diameter, 10 mm thick sample, and an average field of 0.2 T was applied to the 30 mm diameter, 2.5 mm thick sample. The 10 mm diameter probe exhibited a much smaller decrease in resistance and a smaller voltage increase than the 30 mm sample (see Figure 6 and Figure 7).

Furthermore, the 10 mm probe showed a voltage of 0.1 V at 380 K, while the voltage for the 30 mm sample approached 0.3 V at the same hot-side temperature without the application of a magnetic field. See Figure 6.

From Figure 6 and Figure 7, it can be concluded that the 10 mm sample is not suitable for the transducer design; thus, the 30 mm-diameter sample was selected for further analysis.

Applying a magnetic field inside the pill results in a substantial reduction in dynamic drain resistance compared to the non-magnetized sample. Consequently, thermoelectric power and efficiency are observed to improve markedly due to lower dynamic drain resistance for the identical sample, suggesting that applying a magnetic field should be a viable method to enhance the performance of Na_x_CoO_2_ based thermoelectric devices. Under an average axial magnetic field of 0.2 T inside the 30 mm-in-diameter sample, applied in the direction of temperature gradient, there is a significant enhancement in voltage, beginning at 0.13 V at 300 K, rising slowly to 0.2 V at 320 K, and soaring to approximately 0.45 V as the temperature approaches 340 K. See Figure 7. This notable rise in voltage in the presence of a magnetic field is accompanied by the anisotropic magnetoresistance (MR) effect displayed by Na_x_CoO_2_. Axially applied fields sustain a negative MR, while a perpendicular application shifts MR from negative to positive, indicating the suppression of ferromagnetism at lower than 300 K temperatures.

Under an average axial magnetic field of 0.1 T applied along the temperature gradient in the 10 mm-diameter sample, the voltage increased from 0.025 V at 320 K to 0.08 V at 360 K. In comparison, when no magnets were attached, the voltage was 0.015 V at 320 K and 0.05 V at 360 K. Also, it can be noted that at 380 K, while the magnets are demagnetizing, the generated voltage remains the same, 0.1 V. Above 380 K, the NdFeB magnets are completely demagnetized, so no difference in voltage is observed. Still, this transducer’s operating temperature range of 300–380 K is preferred, as it encompasses the water boiling point (373 K), and NdFeB magnets, which are the strongest in the world, offer a maximum remanent magnetic field of 1.5 T (for N55 grade).

**Figure 8:**
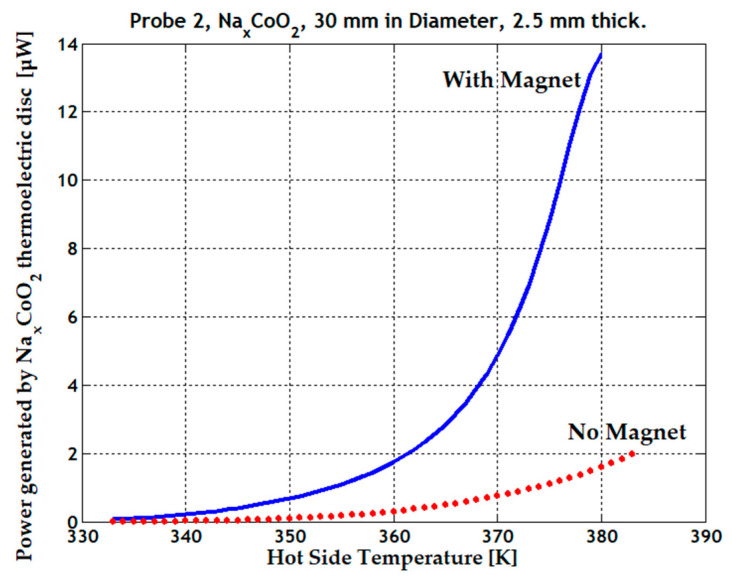
Enhanced generated power (blue line) when the magnetic field is applied to the Na_x_CoO_2_ thermoelectric sample, and the generated power without magnetic field (red dotted line).

The observed electrical behavior, in conjunction with strong electron correlations and lattice frustrations present within the triangular lattice, contribute to substantial thermomagnetic and thermoelectric power, 1.75 µW at 360 K and 13.7 µW at 380 K. See Figure 8. There is a significant power gain with just a 20 K temperature difference.

At 300 K or at room temperature, the sample of sodium cobaltite has a monoclinic cell arrangement; the sodium ion crystallographic position is shifted out from the center of the prism. At room temperature, the crystallographic structure has a lower symmetry and the Na^+^ ions’ motion is also low. As the phase transition occurs near 350 K [22], the crystallographic cells change into a rhombohedral structure, mainly manifesting as a Na ion rearrangement in the interslab to form a higher symmetrical position. The Na mobility will increase as the temperature increases. The motion of Na^+^ in the interslab due to phase shifting creates an additional electrical field that can alter the probe’s voltage response. See Figure 7. Therefore, the presence of an axial magnetic field, the sample hydration, and the phase shifting near 350 K, all allow energy to be harnessed in a more efficient way. The presence of water inside sodium cobaltite leads to its partial autoionization, generating additional ionic charge carriers that are guided by the electric and magnetic fields, along with the usual electronic carriers in the material. In hydrated thermoelectric material, the viscosity of the solution tends to decrease as temperature increases. Lower viscosity allows ions to diffuse more easily, further enhancing ionic and electronic conductivity. In this case, the applied magnetic field also contributes to increased electronic conductivity due to negative magnetoresistance.

In the next subsection, the humidity and water effect is presented. It is expected that the partial autoionization of water in the presence of sodium cobaltite would further improve free carriers’ mobility and conductivity, ultimately leading to an increased power factor.

### 3.2. Water or Moisture Effect on Na_x_CoO_2_

Water or moisture from the air increased the CoO_2_ layer thickness, because the hydrogen atoms of hydronium ions H_3_O^+^ were attached to the oxygen atoms inside the CoO_2_ layers. This bond was creating a tensile strain of the interlayer distance, from which Na atoms can go through a critical transition, meaning that from this point CoO_2_ lattices can become a set of non-interacting monolayers [21]. When hydration time reaches or exceeds 10 days, two other phases are formed, one with four water molecules and another with six water molecules. While one hydrogen bonds to an oxygen atom in the CoO_2_, each oxygen atom and second hydrogen lies in a plane between the Na and Co layers. Such intercalation of four and six water molecules largely separates the CoO_2_ layers and expands the c-axis parameters. To be noted that the distance between crystal layers “c” increases from c = 11.15 Å, for non-hydrated sodium cobaltite to the longest value c = 22.38 Å, for the hydrated phase with six water molecules.

However, these configurations with four and six water molecules are not stable for long at room temperature [29]. The water insertion between alternately stacked CoO_2_ layers and Na layers creates a thermo-chemical battery-like structure. This thermo-chemical battery is based on the deintercalation or intercalation process of Na^+^ ions [30]. One major characteristic of this thermo-chemical battery is that it can store thermal energy as chemical energy, and it can generate ionic currents under the temperature difference. This combination of the accumulation and the generation of electricity could be very useful to integrate into small power sources. The same process can be used to create fuel cells and supercapacitors [31,32], where the sodium cobaltite is selected as electrode material. The materials showed a significantly enhanced surface capacitance of 5.69 F cm^2^. At or above room temperature, the chemical structure of hydrated Na_x_CoO_2_ evolves into Na_x_(H_3_O)_z_(H_2_O)_n_CoO_2_, featuring a mix of Na^+^ and H_3_O^+^ ions interspersed with water molecules, within the CoO_2_ layers. Here z is the count of the total number of hydronium atoms and n is the count of the total number of water atoms. The water is partially ionized. This unique configuration significantly bolsters electrical conductivity due to the increased ion count. To be noted is that the configuration from Figure 1 and Figure 3 was used in all measurements regarding the sodium cobaltite sample hydration. Also, the 30 mm-in-diameter sodium cobaltite sample was manually hydrated with three dosages, 1 mL, 1.5 mL, and 2 mL, by using a syringe. Experiments showed that an optimal hydration dosage of 2 mL increased water evaporation time without damaging the probe, and this dosage was used in all subsequent experiments.

The electrical resistivity variation with the temperature of the hydrated sample, placed in a constant magnetic field (see Figure 1 and Figure 3), was recorded with an HMC8012 Bench Digital Multimeter, produced by Rohde & Schwarz GmbH, Munich, Germany.

**Figure 9:**
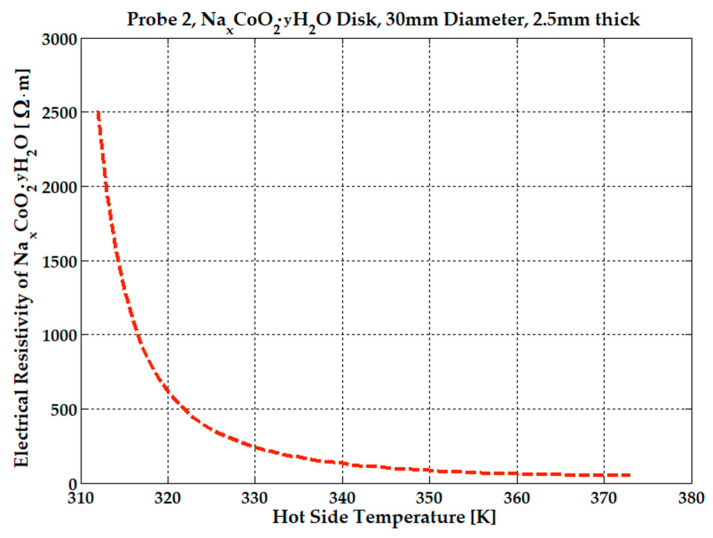
Electrical resistivity of the Na_x_CoO_2_ hydrated sample in a 0.2 T average magnetic field.

At 312 K, the electrical resistivity of the hydrated probe is 2500 Ω·m, at 322 K is 500 Ω·m, and it then decreases to 50 Ω·m at higher temperatures, 360–370 K. See Figure 9. The hydrate formula ${Na}_{x}CoO_{2}\cdot yH_{2}O$ is calculated by dividing the number of moles of water by the number of moles of ${Na}_{x}CoO_{2}$.

The incorporation of water and the application of a magnetic field are projected to escalate these values further. In pristine Na_x_CoO_2_ samples with x > 0.8, the addition of water markedly surges the current from 0.5 μA to a peak of 320 μA at 340 K, enhancing power output by 15 times. However, at temperatures reaching 370 K, rapid water evaporation diminishes power output sharply within three minutes. A viable strategy to mitigate this involves operating at temperatures below 340 K and leveraging the temperature change on the cold side to maintain an optimum temperature gradient.

**Figure 10:**
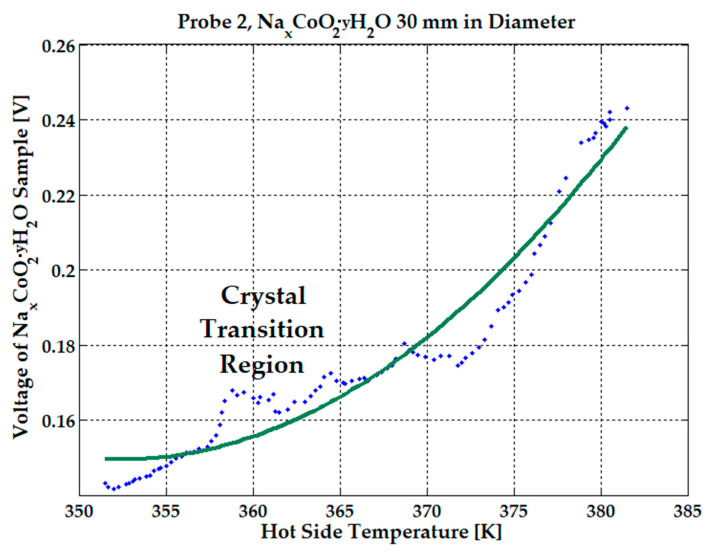
Voltage variation with hot-side temperature of Na_x_CoO_2_ hydrated sample in a 0.2 T average magnetic field. The blue dotted line represents the experimental data and the green solid line represents the interpolated data.

Although voltage increases with temperature from 0.13 to 0.24 V over a 310–380 K temperature range (see Figure 10), the observed peak idle current was between 1.1–1.8 mA for temperatures around 350–360 K. When the water starts to evaporate over 370 K, the measured current drops rapidly down to 74 μA. The estimated power of 200 µW at 380 K can be maintained for several seconds only. See Table 1.

The formation of hydrogen bonds within sodium cobaltite is explained in the next paragraph. Hydrogen bonds are a type of dipole–dipole interaction that occurs between an electronegative atom (such as oxygen) and a hydrogen atom bonded to another electronegative atom. In the context of sodium cobalt oxide, the lattice oxygen atoms are highly electronegative, making them prime sites for hydrogen bonding with water molecules. When water molecules intercalate into the sodium cobalt oxide lattice, they form hydrogen bonds with these lattice oxygen atoms. This can be represented by

H_2_O + O_lattice_ →H–O_lattice_ + O–H, here H–O_lattice_, (1)

which represents the hydrogen bond formed between the water molecule’s hydrogen and the lattice oxygen. The water molecule intercalation has an impact on sodium cobaltite material properties. Hydrogen bonding can alter the electronic environment of lattice oxygen atoms, affecting the material’s electronic band structure and electronic conductivity. Intercalated water molecules can scatter phonons, which are heat-carrying particles, thereby reducing thermal conductivity and improving the thermoelectric figure of merit (ZT). Additionally, the introduction of water molecules and the formation of hydrogen bonds can influence charge carrier concentration and mobility, leading to large variations in the Seebeck and thermomagnetic (if magnets are added) coefficients, even for small temperature differences between the hot and cold sides. Water molecule intercalation increases the electrochemical reactions of NaCoO_2_. The insertion of water molecules enhances the mobility of Na^+^ ions within the layers, thereby improving ionic conductivity, which can be quantitatively assessed using electrochemical impedance spectroscopy (EIS) by measuring the decrease in charge-transfer resistance in the presence of water [15]. Water’s role in modifying the electrochemical stability and cycling performance of NaCoO_2_ electrodes in sodium-ion batteries is critical; it can either facilitate sodium ion transport, improving charge/discharge rates, or induce structural instability, leading to capacity fade, with cyclic voltammetry (CV) and galvanostatic charge–discharge tests being instrumental in evaluating these effects [33,34].

**Figure 11:**
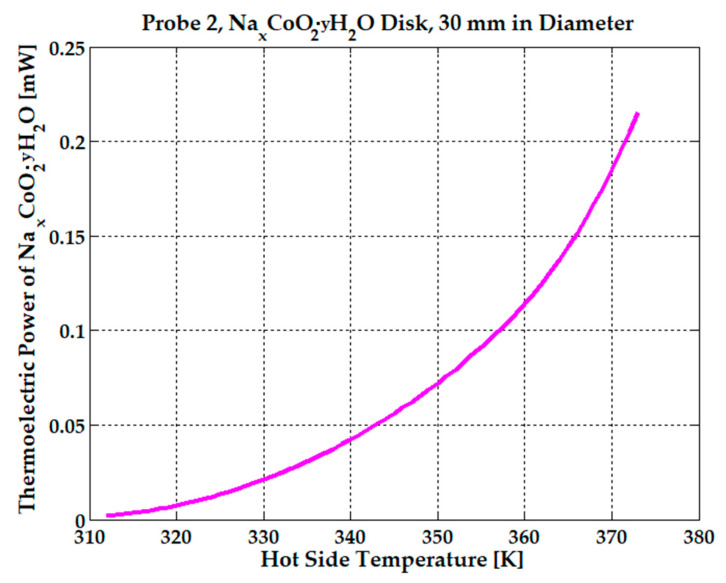
The effect of water on thermoelectric power for the Na_x_CoO_2_ sample placed in a 0.2 T average magnetic field.

Thermal and chemical stability of hydrated NaCoO_2_ can be monitored by various techniques. Differential scanning calorimetry (DSC) and thermogravimetric analysis (TGA) can be used to study the thermal behavior of hydrated NaCoO_2_, helping determine the temperatures at which water is released from the structure, indicating the stability of the hydrated phases [35]. Long-term exposure to moisture can lead to the leaching of sodium ions and oxidation or reduction of cobalt in the lattice, which can be monitored by using chemical analysis techniques such as inductively coupled plasma mass spectrometry (ICP-MS) to track elemental composition changes over time [18]. Sodium cobaltites have been used as electrolytes for fuel cell applications. A fuel cell can be fabricated from a Pd/NaCo_2_O_4_/NaCo_2_O_4_ disk and placed between two sheets of platinum mesh current collectors at anode and carbon paper current collectors coated with Teflon and Nafion [36]. An increase in thermomagnetic and thermoelectric power is observed in Figure 11, with values of 50 µW at 340 K and 100 µW at 357 K, when a quantity of 2 mL of water is injected into the 30 mm-diameter sample. The power doubles with a temperature difference of just 17 K.

**Figure 12:**
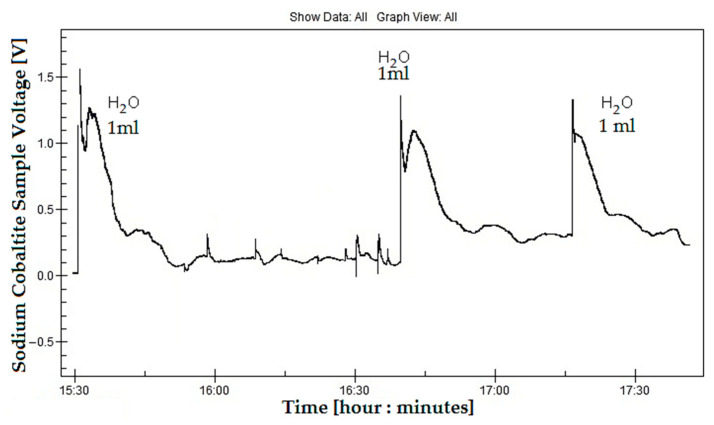
The effect of water and of crystallographic phase transition on the thermoelectric and thermomagnetic voltage for the Na_x_CoO_2_ sample, placed in a 0.2 T average magnetic field and heated to 350 K.

To be mentioned again is that the 30 mm-in-diameter sample was manually hydrated with the optimum dosage of 2 mL by using a syringe, and placed in the electrical heat-press, for all experiments presented above. A peak voltage of 0.24 V was obtained at 380 K and 0.17 V at 360 K as the probe cooled to ambient temperature. The current dropped from 1.8 mA to 0.82 mA in just 2 min, when the temperature was increased from 350 K to 360 K but, after that, the current gradually decreased from 0.82 mA to 0.2 mA in 4 min, until the injected water evaporated. Upon sample cooling from 360 K (current was 0.2 mA) to 350 K, the current slowly reached 100 μA after another 10 min. All graphs from Figure 9, Figure 10 and Figure 11 were realized by letting the sample cool to ambient temperature. An average current of 0.82 mA that lasts about 6 min can be considered at 360 K to be a hot-side temperature. To demonstrate the reliability and durability of the proposed device under hydration conditions, the effect of adding 1 mL, 1.5 mL, and 2 mL of water was monitored for several hours, with 42 mm-diameter NdFeB magnets attached to the sample. These experiments were repeated several days per week for over three months to determine whether the resulting voltage and current data were conclusive. In Figure 12, voltage variation following the addition of 1 mL of water was recorded over several hours. Each time water was added, the voltage spiked to 1.4–1.5 V for a brief period (seconds) before dropping to 1.1–1.2 V and gradually decreasing to 0.3 V over a period of 12 to 15 min. It took approximately 23–30 min for the voltage to stabilize at its lowest value, 0.2–0.3 V, at a constant temperature of 350 K, remaining steady until the injected water evaporated and only ambient humidity remained.

Here, y is the number of moles of water per moles of sodium cobaltite. Experimental observations indicate that an optimal sodium concentration of approximately x ≈ 0.88 significantly enhances the thermoelectric performance of Na_x_CoO_2_ over a hot-side temperature of 370 K. This enhancement could be further amplified by substituting sodium ions with holes, where holes will be replaced by H_3_O^+^ ions obtained from water autoxidation in the cobalt oxide layers, providing a route to increase the power factor. It should be noted that a small fraction (1–10%) of the H_3_O^+^ ions replace Na^+^ ions, while the majority of hydronium ions attach to the oxygen atoms within the CoO_2_ layers. For temperatures higher than 370 K, the figure of merit ZT increases from 0.01 to 0.06.

### 3.3. The Electronic Voltage Booster

**Figure 13:**
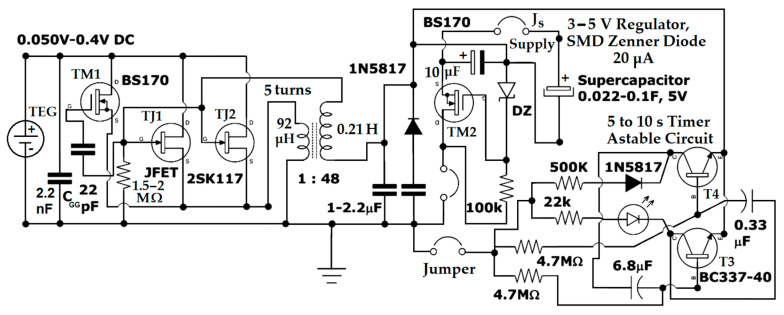
The electronic scheme used for the sodium cobaltite thermoelectric modules, which comprises a Meissner oscillator, a voltage regulator for supercapacitor charging, and an astable timer circuit.

The electronic scheme shown below is used to capture useful electrical energy from five or more thermoelectric modules made of Na_x_CoO_2_ pills stacked and connected parallel. For this parallel connection, it is assumed that all thermoelectric modules are identical, resulting in no variation in output voltages. The electronic device of energy harvesting type is based on the properties of hydrated sodium cobaltite, placed in a magnetic field. A single Na_x_CoO_2_ thermoelectric pill generates 15–20 mV with small temperature differences of 10–12 K between the hot and cold side. The maximum current generated by a single sodium cobaltite sample at a temperature of 340 K is 350 µA. The TJ1 and TJ2 JFET 2SK117 (see Figure 13) transistors used in the circuit can initially conduct and open with a gate-source voltage (VGS) of −0.1 V and a current of 1.5 mA; with only −0.05 V gate-source voltage, the current required to open the transistor is at least 2–3 mA.

It is obvious that the thermoelectric generator (TEG) can be used also in cold weather, when the air temperature is only 280 K or less. The most important fact is that, in any situation, we should have at least a 30 K difference between the hot and cold side to obtain a voltage of 50 mV. In addition, from two to four TEGs could be connected in series to obtain more voltage. As can be observed, each TEG will need no more than 10 pills to make the voltage booster or the Meissner circuit work.

From the datasheet of the 2SK117 JFET transistor, all parameters can be found: variation of drain-source V_DS_ voltage, drain current I_D,_ and all the gate-source voltage (V_GS_) conditions. In case of higher input voltage of V_GS_ being around −0.3V…−0.4 V, the input current can be less than 0.2–0.1 mA.

In the schematic, there are two 2SK117 TJ1 and TJ2 transistors in parallel to share the input current (the JFET transistor is not overloaded in current) and to halve the dynamic drain resistance. Lower resistance also proportionally reduces conduction losses. In conduction, the 2SK117 JFET transistors have a dynamic drain resistance of 50 Ω, while for the 2SK170 JFET, the dynamic drain resistance is between 50 and 150 Ω. For any transistor used as a switch, it is preferable that dynamic drain resistance is minimal to reduce conduction losses. The BS170 MOSFET TM1 transistor has exactly this role of taking over the current in the circuit (it closes) and significantly reducing conduction losses (1.8–5 Ω dynamic drain resistance) when the inductive voltage pulse initially produced by the opening of the JFET transistors is sufficiently high at the gate, starting from 0.8 V and up.

The capacitor between the two gates prevents the simultaneous opening and closing of the transistors, creating a slight delay that allows the inductive pulse to propagate for a determined duration in the transformer’s secondary. When the gate voltage is sufficiently high (>0.8 V), the TM1 BS170 MOSFET transistor takes over the Meissner circuit oscillation up to a maximum voltage of 18 V. The oscillation frequency of the Meissner circuit is mainly determined by the inductance or reactance of the secondary and the capacitance of the capacitor between the TJ1 and TJ2 JFET gate and the TM1 BS170 MOSFET gate, if this is greater than the internal parasitic capacitance of the BS170 and 2SK117 transistors.

**Figure 14:**
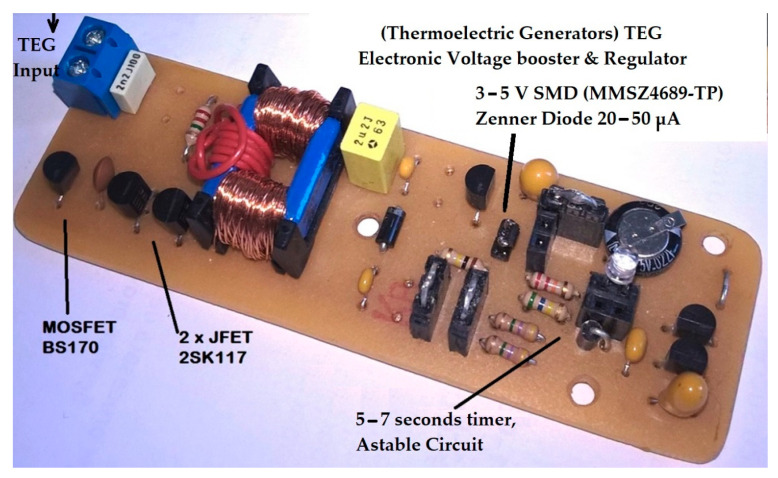
Voltage booster circuit board with Meissner oscillator, 50–400 mV input, and 3–5 V regulator for supercapacitor charging, practical implementation.

The internal parasitic capacitance of the transistors (C_GS_) is sufficiently large, 62 pF. Because the capacitance $C_{GG}=22pF$ between TJ1 and TM1 gates is summed with C_GS_ and TJ1 input capacitance of 13 pF at saturation, resulting in 97 pF, the oscillating frequency will be around 35 kHz. At this oscillation frequency and with a transformer ratio of 1:48, a maximum gate voltage of 18 V can be reached with an input voltage of 0.4–0.5 V (see Figure 13 and Figure 14). Proportionally, with a voltage of 0.2 V, the maximum output voltage obtained on the secondary is 8 V. Also, for an input voltage of 0.1 V, the maximum output voltage will be 4 V.

The 1–2 µF filtering capacitor placed between the secondary of the transformer and ground also serves to limit the voltage to a convenient value for the voltage regulator part.

Since the 1–2 µF filtering capacitor is connected in a series with a combined capacitance of 97 pF, the smaller capacitance (97 pF) will be considered in the Meissner circuit and will be used to determine the oscillating frequency.

It is known that a voltage regulator is more efficient when the input voltage is closer to the output voltage. The capacitor must also be chosen based on the current provided by the thermoelectric generator (TEG) at the transformer’s output; the lower the current, the smaller the capacitor can be and, conversely, the higher the current provided by the generator, the larger the capacitor required for voltage limitation at the output.

For a thermoelectric generator (TEG) with 10 sodium cobaltite pills of 30 mm in diameter and thickness of 2.5 mm, the current at the output of the transformer secondary is 3.5 mA/48 = 73 µA. So, the current drops under 100 microamperes when it reaches the voltage regulator part, with the voltage being limited to 4 V by the respective capacitor (at 0.1 V TEG input). For such small currents, a classic Zener diode would not function correctly and, moreover, all generated power would be consumed by the diode. To prevent a Zener diode from consuming too much of the power generated by the thermoelectrics, the operating current of the diode needs to be as low as possible. Current technology has achieved a 50 microamperes operating current (MMSZ4689-TP SMD type Zener diode used for 5.1 V in all electronic schematics, a component produced by Vishay Semiconductors Company, Shelton, United States).

The diode can operate even below that operating current, down to 20 µA, but in that case, the voltage at the output of the regulator drops below 4 V. The TM2 BS170 MOSFET transistor, a component produced by Infineon Technologies AG, Neubiberg, Germany, used also in the voltage regulation part requires very low gate currents (10…50 µA; the gate of a MOSFET is voltage-controlled) and can support maximum currents of 500 mA between the drain and source.

The 100 × 30 mm electronic board can be further reduced geometrically if all the components are available in the SMD variant. See Figure 14. The 2SK117 JFET transistor from the electronic scheme, a component produced by Toshiba Corporation, Tokyo, Japan, can be replaced by the equivalent 2SK209 SMD, but the transformers are not available in sizes smaller than 10 × 10 × 10 mm. Even if one can be found, very small transformers are wound with very thin winding wire of 0.05mm at a 1:48 ratio, which implicitly increases the dynamic drain resistance of the coil and total conduction losses. A larger transformer with a 15 × 25 mm magnetic core is preferred because it is wound with 0.2–0.3 mm wire; this way the conduction losses are minimized. The same issue occurs with the coil used in switching voltage regulators; it cannot be significantly reduced in size because two essential conditions would no longer be met: inductance greater than 1–10 mH and dynamic drain resistance below 1 Ω.

The electronic board also contains an astable circuit that signals with a red (or yellow) LED every 5–10 s. The high resistance of 4.7 MΩ and 500 KΩ used together with a Schottky diode 1N5817 on the opposite side of the LED minimize energy consumption and allow monitoring of the supercapacitor’s charge level. The red (or yellow, or white) LED can also light up when the voltage on the supercapacitor reaches 1.8 V, 2.2 V, or 3 V. A red LED, without a current-limiting resistor, connected directly to the supercapacitor (the circuit also has a parallel socket for connecting two pins to the load) at a voltage of 3 V, stays lit for at least 1 min.

The estimated rate of water evaporation at 360 K was approximately 4 mL/h, given a specific surface area of 7 cm^2^ and a relative humidity of 40%. At 360 K, an average current of 0.82 mA was recorded (see Table 1) over a 6 min period, with a voltage reaching 0.165 mV. However, water evaporated more quickly, completing in 16 min (compared to the estimated rate of 2 mL per 30 min). Therefore, the current of 0.82 mA was sustained for 6 min, followed by a lower average current of 0.14 mA for an additional 10 min. At 350 K, the current and voltage were sustained for 23 min after 1 mL of water was injected into the sample. The voltage spikes suggest that a sudden current drop occurs simultaneously, keeping thermoelectric power within the same range. However, the crystallographic phase transition at 350 K requires further analysis.

At 340 K, the estimated rate of water evaporation under the same conditions was lower, at 1.7 mL/h. Since the 30 mm-diameter pill was hydrated with 2 mL of water, the estimated time until evaporation was 70 min. Experiments recorded an average current of 0.35 mA (see Table 1), sustained for 35 min. Each pill will require an additional 1.7 mL every half hour to function properly.

## 4. Discussion

The thermoelectric characteristics of Na_x_CoO_2_ have been comprehensively assessed over a temperature range from 300 K to 400 K. Experiments revealed that introducing a magnetic field significantly boosts the voltage and power output, optimizing the material’s thermoelectric performance, as long as the Neodymium magnet’s Curie point is not exceeded. Beyond this temperature, the magnetic field enhancement ceases as the magnetic material undergoes demagnetization and, consequently, the thermoelectric efficiency declines. This observation highlights the temperature-dependent magnetocaloric effect on the thermoelectric properties of Na_x_CoO_2_. Neodymium magnets can be replaced by Grade 26 SmCo Disc Magnets, which have a slightly lower remanent magnetic field of 1.05 T instead of 1.2 T (for N35 magnetization strength), and a comparable coercive magnetic field intensity of 795 kA/m, instead of 868 kA/m. The most important feature of Grade 26 SmCo magnets is the high operating temperature, up to 623 K. SmCo magnets will be used when the thermoelectrical modules are redesigned for temperatures higher than 400 K. In this case, water can no longer be used, so a number of 50 sodium cobaltite pills should be added to the proposed device, to increase the current from 30 µA to 1.5 mA at 420 K. The dry sodium cobaltite samples measured at 300 K exhibited electrical resistivities of 1–2 mΩcm, which were 10–20 times higher than the resistivities measured at 30 K (0.1 mΩcm) [10]. Compared to prior research, samples from this present paper showed poorer conductivity, although their resistivity decreased with increasing temperature. Specifically, the dry sodium cobaltite samples showed resistivities ranging from 447 KΩm at 330 K to 388 KΩm at 340 K, indicating semiconductor behavior. With magnets attached to the dry samples, the resistivity dropped even more sharply, from 90 KΩm at 330 K to 56 KΩm at 340 K. Despite the high resistivity, the probes exhibited a significant voltage increase, from 0.2 V at 320 K to 0.45 V at 340 K. This pronounced rise in voltage with temperature makes the material promising for integration into active transducers. As conductivity reduces, the conduction band level will be much higher than the Fermy energy level. As a result, a large thermopower coefficient is expected, as the experiments displayed. The exact values cannot be determined until we clearly establish the potential electrochemical influence. The phase transition that occurs near 350 K is leading to a crystallographic cell change from a monoclinic to rhombohedral structure. As the Na ions are rearranging to form a higher symmetrical position, some of the Na ions will be expelled from the structure to be replaced by hydronium ions. The hydronium ions are the result of water autoxidation and moisture that is present in the surrounding air. Even though excessive moisture and temperature can temporarily modify the Na ions’ content inside the Na_x_CoO_2_ sample, the incorporation of water within the material’s structure can markedly boost the electrical current and increase power generation. Overall, hydration has a beneficial impact on the material’s thermoelectric response. These results indicate a synergistic potential for combining magnetic fields and material hydration to maximize the thermoelectric power output of Na_x_CoO_2_, paving the way for future advancements in energy conversion technologies. The resistance of the Na_x_CoO_2_ sample to environmental conditions can be enhanced by a mixture of Ca_3_Co_4_O_9_–Na_x_CoO_2_ interlayers [18]. Other solutions can be adopted, like doping the samples with no more than 10% sodium silicate (waterglass) or by doping the sodium cobaltite with heavy metal oxides [16]. The presented electronic device can capture useful electrical energy from five or more thermoelectric modules made of Na_x_CoO_2_ samples stacked and connected parallel, when the pills are hydrated. It was assumed that all thermoelectric modules were identical; no cell output voltage change was noticed.

This thermoelectric active transducer cannot function without an electronic circuit and an additional current boost. In this study, an electronic device of energy harvesting type, based on the properties of hydrated sodium cobaltite and placed in a magnetic field, is presented. The Meissner oscillator or electronic booster can either operate at lower than 50 mV input voltages, requiring a current for TJ1 and TJ2 2SK117 JFET of 2–3 mA at the input, or can be supplied by multiple thermoelectric modules mounted in a series to reach 0.1–0.2 V and a current of 0.2–0.5 mA. Calcium cobaltites and sodium cobaltites have the ability to work as humidity and temperature sensors. Due to significant resistance and current variation, resistance drops from 380 KΩ (for a dry probe with magnets) to 815 Ω at 330 K, when the sample is hydrated and placed in a magnetic field. This indicates that the sensitivity of sodium cobaltite humidity sensors is very high. The humid environment will also increase the current until the transducer becomes autonomous. Additionally, when the sample is hydrated and placed in a magnetic field, the voltage increases from 0.14 V at 340 K to 0.24 V at 380 K. Furthermore, the voltage variation in a dry sample with magnets attached, ranging from 0.2 V at 320 K to 0.45 V at 340 K, suggests that the dry sample is very sensitive to temperature. The sodium cobaltite sample is much more efficient at converting thermal energy into useful electrical energy and works as a thermo-chemical battery. The hydronium ion intercalation between stacked CoO_2_ layers and Na layers creates a thermo-chemical battery-like structure. Electrochemical reactions and the heat transfer are based on the deintercalation or intercalation process of Na^+^ ions. As the number of freely moving Na^+^ ions increases in the aqueous solution from the process of deintercalation, a small decrease in sodium concentration from 0.07 to 0.1 is observed [21,33]. The rate of sodium concentration decrease depends on water quantity in ml, and is about 0.01 per ml. Because the sodium cobaltite pill optimum hydration is for 2 mL of water injection, an increase of sodium concentration to 0.9 must be considered to compensate the process of sodium deintercalation from the sodium cobaltite structure.

The advantage of this thermo-chemical battery is that it can store thermal energy as chemical energy. Further, the additional ionic current can stimulate and boost the number of electrons under higher temperature differences and higher applied magnetic fields. The same process of sodium ions co-intercalation can be used to create fuel cells [36] and supercapacitors [31,32]; in both cases, the sodium cobaltite will be used as electrode material.

## 5. Conclusions

The research presented in this paper explored the effects of temperature, hydration, and magnetic fields on the thermoelectric performance of sodium cobaltite (NaxCoO2) samples, focusing on optimizing the material for energy harvesting applications. The material can also be used for humidity and temperature sensing. Hydration of the sodium cobaltite samples played a crucial role in improving their thermoelectric performance. Water molecules, upon intercalating into the CoO_2_ layers, created hydrogen bonds that enhanced ion mobility, increasing the current output and decreasing resistivity. The results showed that hydrated samples with attached NdFeB magnets produced a significant power increase, reaching up to 135 µW at 360 K when water was injected. However, at higher temperatures (above 370 K), water evaporation limited sustained power output. The application of a strong axial magnetic field significantly reduced the material’s resistivity and boosted voltage output. For example, applying an average magnetic field of 0.2 T resulted in a notable increase in voltage from 0.2 V at 320 K to 0.45 V at 340 K. The thermomagnetic effect induced by this magnetic field enhanced electron mobility, thus improving overall power generation. The phase transition near 350 K from a monoclinic to a rhombohedral crystal structure improved Na^+^ ion mobility, contributing to the material’s thermoelectric response. This transition response was further enhanced by hydration and magnetic fields. To improve the stability of sodium cobaltite in excessively humid environments, this study suggests two solutions: interlayering with calcium cobaltites; or incorporating small percentages (0.05%) of dopants like Ca or Ba to substitute Na in the lattice structure. Because Ba has the largest atomic radius, its characteristics can impact the electronic band structure, enhancing conductivity and expanding the lattice to create more room for hydronium ion intercalation. Substituting Na with Ba may also improve the thermoelectric performance of sodium cobaltite by altering charge carrier density and phonon scattering, which affect the Seebeck coefficient and thermal conductivity. Sodium cobaltite-based thermoelectric modules showed potential for use in small power generation systems and as transducers for sensing temperature and humidity. A custom-designed electronic circuit (Meissner oscillator) was successfully developed to amplify the voltage output of these thermoelectric elements, which were mounted in parallel, making them function like a thermoelectric battery. The materials could also be adapted for use in supercapacitors, or fuel cells, due to their ability to store energy thermochemically and convert it into electrical power under temperature gradients.

This study aimed to optimize the hydration process, identifying 2 mL as the optimal dosage for 30 mm-diameter samples, and refining the device design to sustain power output over longer periods, especially in environments with fluctuating humidity.

Further research could explore higher operating temperatures by replacing neodymium magnets with samarium-cobalt (SmCo) magnets, which have a higher Curie temperature. This could extend the operational temperature range beyond 400 K; however, the entire device would need to be redesigned to withstand these temperatures. Further research should also investigate operating temperatures between 200 K and 300 K, using the strongest neodymium-iron-boron magnets (N55) available on the market, attached to a sample immersed in freezing water.

## Figures and Tables

**Table 1:** Dry and hydrated (2 mL of water) Na_x_CoO_2_; 30 mm-in-diameter sample placed in a 0.2 T average magnetic field.

P type Material	Dry Na_x_CoO_2_ (Magnet)	Hydrated Na_x_CoO_2_ (Magnet)
Voltage [mV]—340 K	450	143
Current [µA]—340 K	0.5	350
Power [µW]—340 K	0.22	50
Voltage [mV]—360 K	1120	165
Current [µA]—360 K	1.6	820
Power [µW]—360 K	1.8	135
Power [µW]—380 K	13.7	>200

## Data Availability

The original contributions presented in the study are included in the article, further inquiries can be directed to the corresponding author.
